# Optical force estimation for interactions between tool and soft tissues

**DOI:** 10.1038/s41598-022-27036-7

**Published:** 2023-01-10

**Authors:** Maximilian Neidhardt, Robin Mieling, Marcel Bengs, Alexander Schlaefer

**Affiliations:** Institute of Medical Technology and Intelligent Systems, Hamburg University of Technology, Am Schwarzenberg-Campus 3, Hamburg, 21073 Germany

**Keywords:** Biomedical engineering, Applied optics

## Abstract

Robotic assistance in minimally invasive surgery offers numerous advantages for both patient and surgeon. However, the lack of force feedback in robotic surgery is a major limitation, and accurately estimating tool-tissue interaction forces remains a challenge. Image-based force estimation offers a promising solution without the need to integrate sensors into surgical tools. In this indirect approach, interaction forces are derived from the observed deformation, with learning-based methods improving accuracy and real-time capability. However, the relationship between deformation and force is determined by the stiffness of the tissue. Consequently, both deformation and local tissue properties must be observed for an approach applicable to heterogeneous tissue. In this work, we use optical coherence tomography, which can combine the detection of tissue deformation with shear wave elastography in a single modality. We present a multi-input deep learning network for processing of local elasticity estimates and volumetric image data. Our results demonstrate that accounting for elastic properties is critical for accurate image-based force estimation across different tissue types and properties. Joint processing of local elasticity information yields the best performance throughout our phantom study. Furthermore, we test our approach on soft tissue samples that were not present during training and show that generalization to other tissue properties is possible.

## Introduction

**Figure 1:**
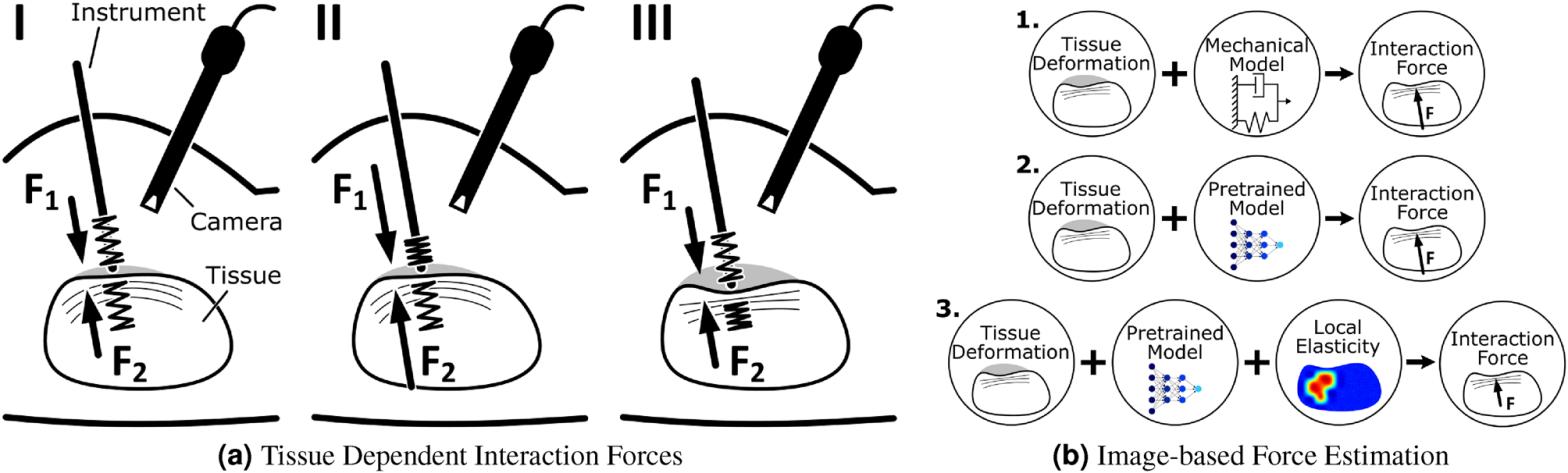
Image-based Force Estimation: **(a)** Abstract illustration of the fundamental problem underlying force estimation without integrated sensors. The springs indicate elastic properties and the camera observes tissue deformations. (I) During tool-tissue interaction, the applied force $F_1$ will deform the tissue and the opposing reaction force $F_2$ will be equal in magnitude. However, the same deformation can be related to greater forces for stiffer tissue (II) or the same force may result in larger deformations for softer tissue (III). Therefore, observing only the deformation will generally not allow estimating the interaction forces if the tissue elasticity is unknown. **(b)** Previous approaches have not considered changes in elastic properties and relied on predefined biomechanical models (1.) or pretrained neural networks (2.) to derive interaction forces from the observed deformation. We instead propose an image-based force estimation model that additionally considers local tissue properties via elastography (3.) and that does not require the material to be known in advance.

Robotic-assisted surgery (RAS) systems such as DaVinci or Senhance are becoming more available in surgical practice^1^ and even less complex medical procedures are performed by RAS, e.g., cholecystectomy and hernia repair^2^. RAS offers a better outcome for the patient by reducing trauma through a minimally invasive approach and results in shorter recovery time^3^. For the surgeon ergonomics are improved during an intervention^4^. However, these benefits come at the expense of the natural haptic perception that surgeons rely on when palpating tissue in open surgery. In RAS, force feedback is associated with shorter operating times, fewer errors during surgery and a reduced mental workload^5^. Force feedback is particularly important for complex procedures and can increase the learning curve for trainees when it is available^6, 7^. Force estimates can also be used to implement safety features that limit forces and prevent soft tissue damage^8^. The lack of real-time force feedback remains a challenge and limits clinical systems in practice^9–12^. Feedback on tool-tissue interaction forces will also be essential for greater autonomy and intraoperative tissue assessment in robotic surgery^13^.

Intuitively, the integration of force sensors into surgical tools, e.g., Bragg sensors, strain gauges and piezoelectric sensors, has been considered and is commonly referred to as the direct approach^14, 15^. Direct approaches offer high accuracy but also have major drawbacks—most notably cost and sensor sterilizability. These limitations have kept direct approaches from widespread clinical application, although research has been ongoing for over 20 years^16^. Alternatively, indirect approaches aim to separate force sensing from the surgical tool, e.g., by considering force models and actuator inputs^17, 18^. Recently, indirect methods for image-based force estimation have attracted more attention^15^, especially machine learning-based approaches. Image-based force estimation aims to derive the tool-tissue interaction forces based on the observed deformation of the soft tissue. However, the relationship between load and deformation is tissue dependent and exclusively observing tissue deformation is generally not sufficient (see Fig. 1a). Previous approaches assumed a predefined material model for each organ but soft tissue properties are highly dependent on the patient and mechanical properties change locally due to pathological conditions^19, 20^, limiting these approaches in practice. Therefore, the question arises of how to adequately account for tissue elasticity in image-based force estimation.

Initial approaches for image-based force estimation used an explicitly defined biomechanical model. Miller et al. proposed a hyper-viscoelastic constitutive model to estimate soft tissue properties for brain tissue^21^. The model was tuned by performing in-vivo indentation experiments. Subsequently, forces on similar brain tissue could be estimated by tracking the position of a tool relative to the tissue. This approach was further adapted by using optical cameras to track the surface of the tissue and then mapping depth values to force estimations. Typically, deformable template matching methods were used to match the measured surface profile to an assumed biomechanical model^22, 23^ Instead of a biomechanical model, the relationship between load and deformation is implicitly learned for deep learning approaches and the trained model is highly dependent on the provided training data. Deep learning approaches with RGB-D images as input have been demonstrated for individual materials with recurrent neural networks^24, 25^ and convolutional neural networks (CNN)^26^. However, the generalization of deep learning models to other material properties has not been investigated extensively. Without the ability to generalize to new samples, training data for all relevant tissue types and pathological stages need to be acquired. Moreover, even with accurate models for different tissues, local changes in material properties demand a more versatile solution that doesn’t depend on the manual selection of models^27^.

We therefore propose to employ optical coherence tomography (OCT) and shear wave optical coherence elastography (OCE) to directly consider tissue properties for image-based force estimation (see Fig. 1b). OCT offers volumetric imaging with high spatial and temporal resolution, enabling elastography and visualization of tissue deformations in a single modality. OCT has been considered for accurate image-based force estimation with single volumes^28^ and 4D temporal sequences as input^29^. Promising results with OCT based force estimation were also demonstrated on xenograft mouse models with vascularized prostate tumors^30^. Additionally, OCE is ideal for local elasticity estimates due to the small field of view (FOV) and its high spatial and temporal resolution. Multiple methods for quantitative OCE with different loading mechanisms have been proposed. Miniaturized compression based OCE can provide estimates at a high spatial resolution but requires complex and sensitive sensors at the tool tip^31, 32^. Instead, we implement shear wave elastography imaging (SWEI), where a shear wave is excited on the tissue surface, e.g., by a piezoelectric element^33^ or an air-pulse^34^, and the high frequency imaging can be used to track the propagating wave. The elasticity of the tissue is directly related to the velocity of the shear wave which can be estimated by detecting the dominant local wavenumber in the frequency domain^35–37^. We combine OCT and OCE, jointly perform data processing with a multi-input deep learning network and estimate tool-tissue interaction forces. We additionally derive surface deformation data from our OCT volumes to demonstrate the advantage of our approach in a case where only surface deformation data is available. Note that our system does not rely on knowing the biomechanical properties of the soft tissue in advance, as suggested in the literature^21–23^.

The main contributions of this work are: (1) Demonstrating the impact of tissue elasticity on image-based force estimation by evaluating deep learning models on elasticities that are not considered during training. (2) Showing that neural networks are able to generalize to unknown materials and demonstrating the advantage of our system that incorporates local elasticity estimates. (3) Combining the findings into a single setup that provides force estimation even when the application is shifted from phantoms to ex-vivo soft tissue samples.

## Methods

In the following, we present our experimental setup with a robot for data acquisition on phantoms with varying elasticity as well as ex-vivo soft tissue and define our deep learning approach.

### Problem definition and data representations

**Figure 2:**
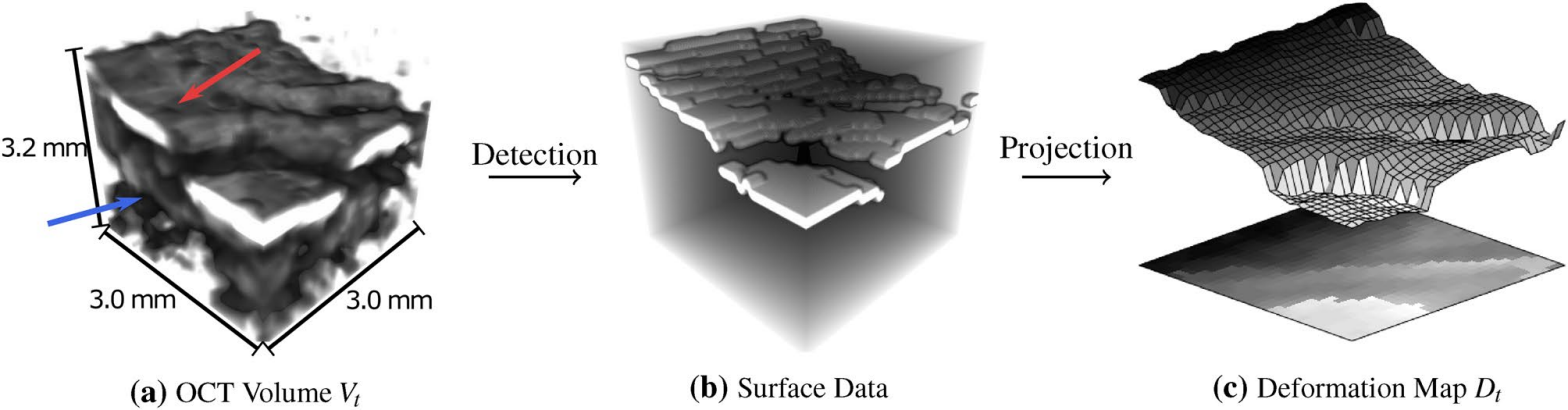
Data Representation: Visualization of the two data representations considered during our learning tasks. **(a)** The volumetric OCT scan $V_t$ contains the surface data (red arrow) and the depth information (blue arrow). **(b)** We detect the sample surface data in the OCT volume. **(c)** We project the surface data onto our 2D deformation map $D_t$. During training, the influence of depth information is analyzed by comparing volumetric data (left) and surface projection data (right).

We consider image-based force estimation for tool-tissue interactions with regard to tissue elasticity. We estimate the axial force $F_t \in {\mathbb {R}}$ at a time step t based on spatio-temporal OCT volume data $V_t \in {\mathbb {R}}^{hxwxd}$. $V_t$ visualizes the deformations caused by tool-tissue interaction in comparison to a reference volume $V_{ref}$. For the observed location *L* on a given sample *S*, the relation between the applied force and the resulting deformation depends on the sample elasticity $E_{S,L} \in {\mathbb {R}}^{hxwxd}$. Prior to the force application, we acquire a sequence of OCT cross-section images $I_{\tau } \in {\mathbb {R}}^{hxw}$ at time step $\tau$ with simultaneous shear wave excitation. We approximate the elasticity at location *L* via the shear wave phase velocity $v_{S,L} \in {\mathbb {R}}$. We further consider an alternative input representation by a projection $P: {\mathbb {R}}^{hxwxd} \rightarrow {\mathbb {R}}^{wxd}$ which maps the sample’s surface in $V_t$ to a deformation map $D_t$. A visualization of the data representations are given in Fig. 2. Our multi-input learning problems are $V_t, V_{ref}, v_{S,L} \rightarrow F_t$ and $D_t, D_{ref}, v_{S,L} \rightarrow F_t$, respectively. We initially regard tissue mimicking gelatin phantoms with seven different elasticities $G_i$. Afterwards, we evaluate our methods on chicken heart soft tissue unseen during training.

**Figure 3:**
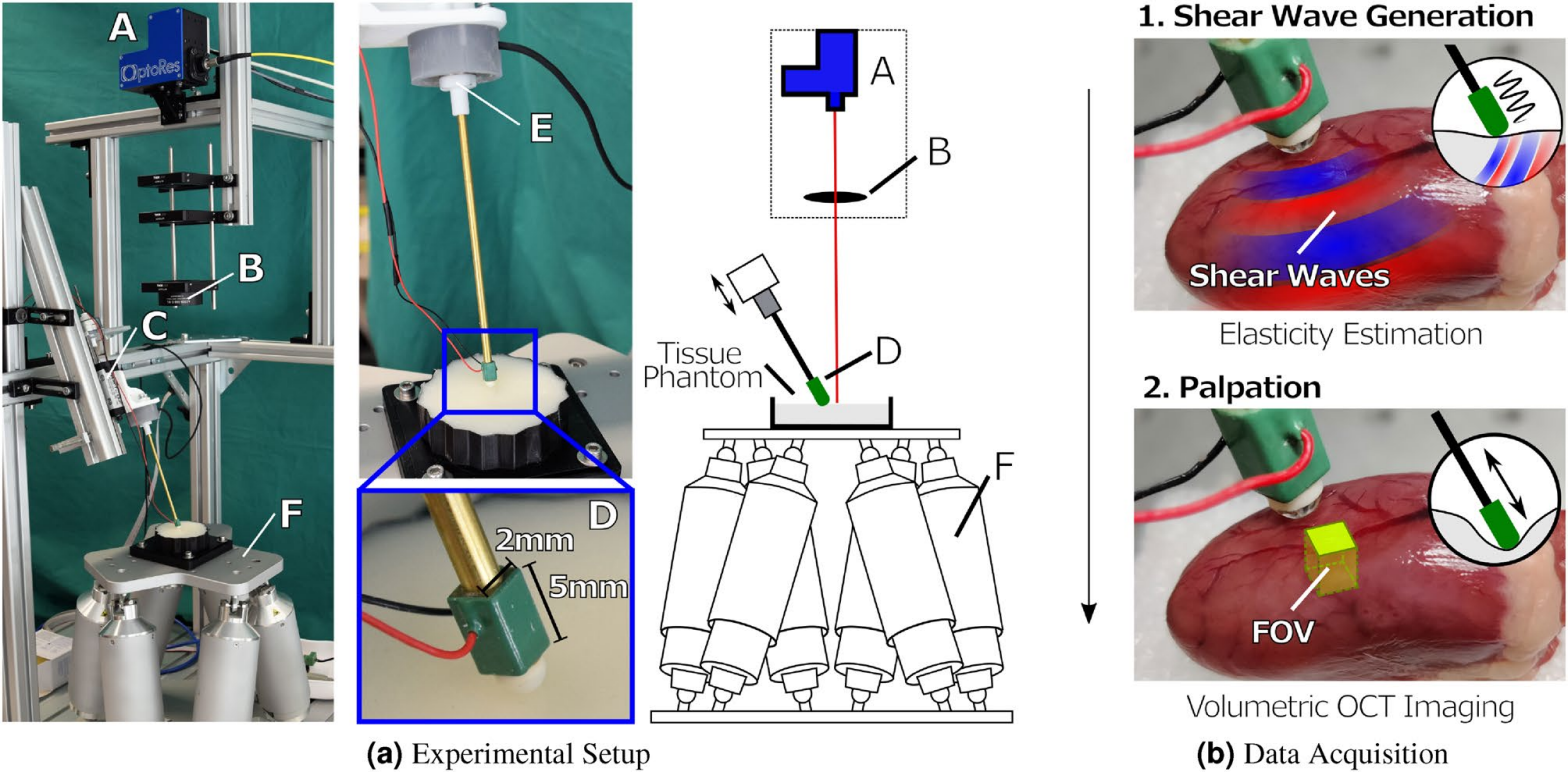
Experimental setup and data acquisition: **(a)** The experimental setup includes a high frequency scan head (A), a lense system (B), a stepper motor (C) which drives the palpation tool fitted with a piezoelectric element (D) along its central axis, a high resolution force sensor (E) for ground truth data annotation and a hexapod robot (F) for positioning the sample. Depicted is the setup with a gelatin phantom. **(b)** For data acquisition, we excite shear waves through vibration of the piezoelectric element (as indicated in red and blue) and OCE data is recorded. During tool-tissue interaction, we acquire volumetric image data as seen on the chicken heart.

### Experimental setup

For data acquisition we present an experimental setup depicted in Fig. 3a. We employ a high-speed swept-source OCT system (OMES, Optores, Germany) with an axial scan rate of 1.5 MHz, a central wavelength of 1315 nm and an axial resolution of 15 $\mu$m in air. A scan head deflects the OCT beam to acquire $2D+T$ SWEI data ($h \times w \times t$) with a spatial resolution of 476$\times$32 pixels (3.5$\times$3 mm) along the depth *h* and lateral axis *w* and a temporal resolution of 14.2 kHz. The same scan head is also used for high-speed volumetric data acquisition ($h \times w \times d$) with a spatial resolution of 476$\times$32$\times$32 pixels (3.5$\times$3$\times$3 mm) and a temporal resolution of 833 Hz. An optical lens system with a focal length of 300 mm is positioned between scan head and tissue. A hexapod robot (H-820.D1, Physik Instrumente, Germany) positions the sample for data acquisition at multiple locations. The robot allows us to move the tissue relative to the FOV of the OCT volumes. Please note, that the FOV relative to the palpation position is fixed. Before data acquisition we drive the robot along the lateral axes *w* and *d* of the volume to the desired location *L*. Next, we drive along the axis *h* of the volume until the surface of the tissue is positioned inside the OCT volume at a depth of approximately 0.5 mm. Surface detection is performed by maximum intensity detection along the depth axis. In our experimental setup design the direction of the robot’s axes correspond to the volume axes. We acquire ground truth for our force data using a high resolution force sensor (Nano 43, ATI, USA) with a temporal resolution of 500 Hz.

### Experimental data acquisition

We prepare seven different gelatin gels $G_i$ with a weight ratio of gelatin to water of 5 %, 7.5 %, 10 %, 12.5 %, 15 %, 17.5 % and 20.0 %. For in-house gelatin preparation we carefully follow a recipe. Titanium dioxide is added to the heated mixture to increase OCT contrast. The phantoms as seen in Fig. 3a have a diameter of 100 mm and a cylindrical height of 10 mm. We manufacture six phantoms for each gelatin gel $G_i$ and acquire data at 9 locations on each phantom. In addition, we record data from 10 ex-vivo chicken hearts at 2 locations. At each location we first estimate the local tissue elasticity (SWEI Data) and subsequently palpate the tissue for the acquisition of force estimation data.

#### SWEI data

Shear waves are excited at the surface of the tissue during high-frequency 2*D* OCT imaging. A piezoelectric element is driven continuously by a sinusoidal signal with a frequency of 1000 Hz for 0.8 s and a peak-to-peak voltage of 210 V. The tip of the piezo is fitted with an epoxy dome to facilitate shear wave excitation inside the tissue, as seen in Fig. 3b, top.

#### Force estimation data

We acquire OCT volumes for image-based force estimation with the piezo element as the palpating tool tip (see Fig. 3b, bottom). First, the tool tip is positioned on the surface of the phantom by carefully driving towards the sample until a force threshold of 0.01 N is exceeded. Second, training data is acquired while driving a sinusoidal profile. The stepper motor is actuated over three cycles with an insertion distance of 2.5 mm and velocities ranging between 0.5mm s$^{-1}$–3mm s$^{-1}$. Additionally, we record OCT data while driving to 20 positions randomly chosen within an insertion distance of 0.5 mm–2.5 mm and a palpation velocity of 2mm s$^{-1}$–7mm s$^{-1}$. The motion represents a pushing task that is commonly performed in minimally invasive surgery^38^. The random palpation data set is used for evaluating the robustness of our methods and is excluded from training.

### Pre-processing

We crop OCT volumes along the depth axis *h* to a length of 200 px and downsample the volumetric data $V_t \in {\mathbb {R}}^{hxwxd}$ to a size of $32 \times 32 \times 32$ pixels for efficient data processing. We assign a force value to each volume by matching timestamps and interpolating the force sensor data. For the 2D deformation map representation $D_t$, we employ a maximum intensity projection along axis *h* for $\forall (w,d) \in {\mathbb {R}}^+$. To ensure surface detection only maximum intensity values above 50% of the mean intensity of the whole volume are utilized, holes in the deformation map are closed by 2D interpolation.

### Shear wave phase velocity estimation

**Figure 4:**
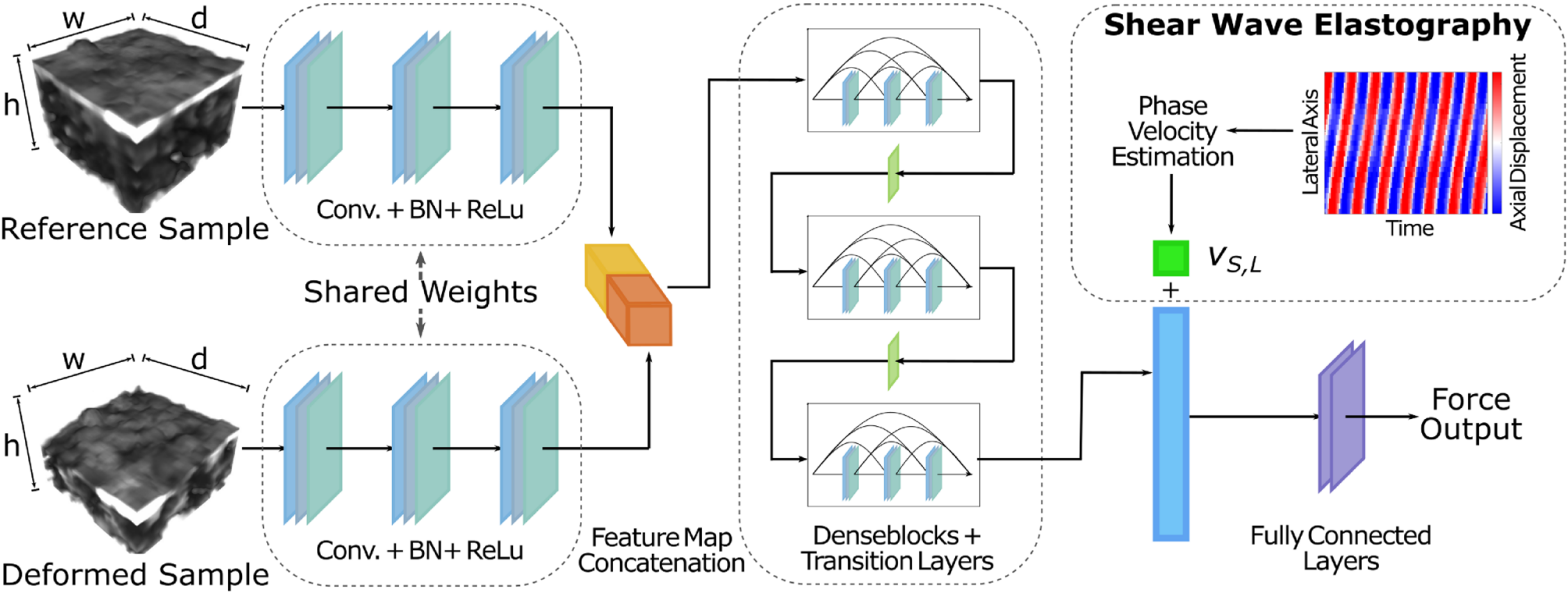
Data Processing: Siamese DenseNet architecture with fusion of SWEI phase velocity. The input and a reference sample are initially processed separately and the obtained feature maps are aggregated. SWEI fusion can optionally be conducted by appending the phase velocity $v_{S,L}$ after the GAP. Convolutional layers employ 3D convolutions for $V_t$ (depicted above) and 2D convolutions for $D_t$. Input sizes $h \times w \times t$ are $32 \times 32 \times 32$ and $1 \times 32 \times 32$ for $V_t$ and $D_t$, respectively.

We crop each 2*D* image to a length of 32 px beneath the surface along axis *h* resulting in an images size $I_{\tau } \in {\mathbb {R}}^{hxw}$ of $32 \times 32$ pixels. We ensure shear wave propagation along the lateral image axis *w*. To estimate the shear wave velocity we unwrap the phase of the complex OCT data at each spatial position along the temporal axis. Next, we take the mean along the depth axis resulting in a 2D space-time representation as shown in Fig. 4, top right. Shear wave phase velocity estimation is performed in the frequency domain similar to^36, 37^. First, we define 30 randomly sampled subsets with a length of 800 time steps. For each subset we evaluate the phase velocity and report the mean of all estimates. We transform the 2D space-time phase data into the k-space by using the 2D discrete FFT. We apply a high-pass filter and an angular sector filter to remove amplitude signals around 0 Hz. To further reduce background noise we apply a threshold filter which removes signals with $<10\%$ of the overall maximum amplitude in the k-space. We determine the index *i*, *j* of the maximum amplitude in k-space and estimate the shear wave phase velocity $v_{S,L} = {f_i}/{k_j}$ with the temporal frequency *f* and the wavenumber *k*.

### Deep learning architectures

We follow the approach of densely connected convolutional networks (DenseNet)^39^. 3D and 2D operations are used for volumetric inputs and surface inputs, respectively. We consider a Siamese architecture where the model is provided with a reference input in addition to each input at time step *t* as depicted in Fig. 4. The reference is acquired prior to sample-instrument interactions for each location and sample with *F* = 0 N. Both input and reference volume are processed within the initial Siamese stage consisting of three convolutional layers. Model parameters are shared for both inputs and the obtained feature maps are concatenated. DenseNet blocks with transition layers follow after concatenation. For 3D kernels, we employ three DenseNet-blocks of 3 layers each and a growth rate of 6. For 2D inputs, we adjust model width and depth to achieve a similar size regarding model parameters. We therefore add an additional DenseNet-Block with a growth rate of 8. Global average pooling layer (GAP) is followed by two successive fully connected layers with one scalar output. We employ the rectified linear activation function^40^. Batch normalization is implemented to provide regularization and to speed up training^41^. The additional SWEI information can optionally be fused into the architecture by appending the phase velocity $v_{S,L}$ to the feature vector after GAP. In the following, our multi-input models combining OCT data with the phase velocity will be denoted 2D+SWEI and 3D+SWEI for surface and volumetric inputs, respectively. Models without the fusion of SWEI information will simply be denoted 2D and 3D with respect to the selected data representation. GPU (RTX 3090, NVIDIA Corporation, USA) inference times are $3.34\pm 30$ ms and $3.30\pm 37$ ms for architectures with surface and volumetric inputs, respectively.

### Training

Our phantom data set consists of 3.7$\times {10}^{5}$ labeled volumes recorded during sinusoidal palpation and 4.5$\times {10}^{5}$ samples acquired during random palpation, equally distributed across all elasticities. For soft tissue, we collect 4.1$\times {10}^{4}$ and 4.3$\times {10}^{4}$ samples for sinusoidal and random palpation, respectively. In general, we train our models with sinusoidal force trajectories and evaluate with random movement exclusively. We train all models using the mean squared error (MSE) as our loss function for 150 epochs with a batch size of 128. Following the one cycle learning rate policy^42^, learning rates between 1$\times {10}^{-4}$ and 1$\times {10}^{-3}$ are used. We use the Adam algorithm with default parameters^43^. Model weights of all convolutional layers are initialized using He initialization^44^.

### Experiments

We perform three experiments: (1) We train our network for force estimation exclusively on a single gelatin gel $G_{i}$ and illustrate the impact of elasticity by applying the model to other gelatin gels $G_{j}$ with $i,j \in 1,2,...7$. (2) We investigate if the models can generalize to elastic properties not included in the training data ($G_i \, \forall G \in {\mathbb {A}} \backslash \{G_j\}$) when training data includes multiple tissue elasticities and evaluate the impact on performance when including local elasticity estimates. (3) Finally, we evaluate our models performance on unknown soft tissue palpation data when trained on gelatin phantom data with multiple elasticities. Our data splits are chosen accordingly. First, we consider the impact of elasticity by training separate models for each gelatin gel. Therefore, we split our data into 6 subsets separated by the different phantoms for each gel. We then consider generalization to new material properties by dividing our data into 7 subsets based on the different gelatin gels. In both cases, we follow a cross-validation scheme where one subset is split into a validation and a test set and the remaining subsets are used for training. Finally, we evaluate our previously trained models on the adaptation from phantom to tissue data. To increase the robustness of the final models, we consider a cross-validation ensemble using the mean as our voting method. Model performance is reported based on the test sets with mean and standard deviation. We evaluate the root mean square error (rMSE) and Pearson correlation coefficient (pCC). As the range of applied forces increases with elasticity, we additionally report the normalized mean absolute error (nMAE), defined as the mean absolute error (MAE) divided by the observed range of forces $F_{G_i}$ for each gelatin gel *i*.

**Figure 5:**
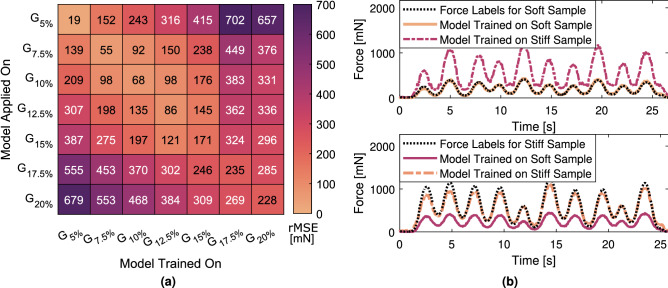
Force estimation: **(a)** Heatmap of the rMSE [mN] of all force estimates for individually trained models with 3D inputs $V_t$. The x-axis denotes the gelatin gel on which the model is trained and the y-axis the gelatin gel used for evaluation. Better performance is given if the applied gelatin gel is similar to the training data. **(b)** Examples that display the impact of elasticity on force estimates when elasticity is not considered. A model trained on “stiff” samples ($G_{17.5\%}$) applied on a “soft” material ($G_{7.5\%}$) overestimates the applied forces due to the large deformations (top). Vice versa, forces are underestimated when transferring a model to stiffer materials (bottom). Line color is based on the rMSE and the colormap in **(a).**

**Table 1:** Experimental Data: Range of minimal and maximum mean surface deformation in px during palpation experiments and deformation relative to the ground truth force given for all experiments performed for each gelatin concentration.

$G_i$ [$\%$]	5	7.5	10	12.5	15	17.5	20
Deformation Range [px]	$5.31\pm 0.64$	$5.34\pm 1.00$	$4.94\pm 0.72$	$5.08\pm 1.02$	$5.67\pm 1.70$	$4.66\pm 1.38$	$6.01\pm 1.74$
Relative Deformation [px N$^{-1}$]	$25.80\pm 4.51$	$12.06\pm 2.28$	$8.31\pm 1.66$	$6.33\pm 1.43$	$5.78\pm 1.75$	$3.56\pm 1.40$	$3.84\pm 1.24$

### Force estimation for individual materials

For visualizing the impact of elasticity, models are initially trained separately for each material Fig. 5a. The shown models do not consider elastic properties via SWEI fusion and are only trained on data from a single gelatin gel. By way of example, results are only displayed for models trained with volumetric inputs. The rMSE ranges from 0.19 to 235 mN for the application on samples from the same material (diagonal of Fig. 5a). Considering the surface deformation, the maximum range of movement and the displacements relative to the applied forces are given in Table 1 for each material. The range of the surface movements is similar for all experiments performed on gelatin phantoms with a mean of 5.28(0.45) px. The surface deformation relative to the applied force decrease for stiffer phantoms correlating with the increase in force estimation errors (pCC$={-0.76}$). Transferring the application to other materials with different elastic properties visualizes the impact of elasticity, resulting in increased errors for the force estimation. Under- and overestimation of the forces is visible for more and less elastic samples, respectively (see e.g. in Fig. 5b). The largest differences in elasticity also correspond to the largest average errors.

### Generalization of force estimation models

**Figure 6:**
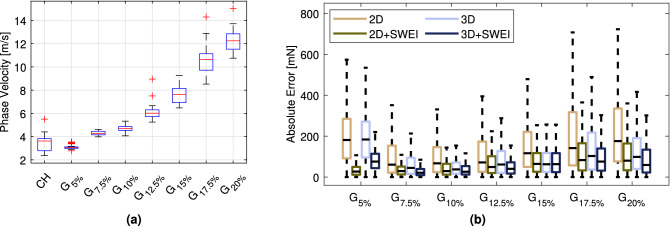
**(a)** Estimated phase velocities in gelatin phantoms and chicken heart (CH) soft tissue. **(b)** Absolute error of the force estimation models generalizing to each sample elasticity. Model performance is compared between 2D and 3D inputs as well as with and without SWEI fusion. Outliers are omitted and colors follow scientifically derived recommendations.^45^

We report the results for models tasked to generalize to elastic properties not present in the training data. We compare the models with only 2D and 3D deformation inputs to our fusion models which additionally consider elasticity via the phase velocity information (2D+SWEI and 3D+SWEI). The velocity estimates from all locations across all samples are displayed in Fig. 6a. Overall, the method displays good differentiation between the different sample types. Within-group variation increases with increasing sample stiffness and phase velocity, especially for 15 % and 17.5 % gels. Regarding model performance, all evaluation metrics for each fold representing a new elasticity, as well as the mean across all folds in Table 2. The absolute errors for the force estimation models are also displayed in Fig. 6b. Considering models without SWEI fusion, 3D inputs clearly outperform the 2D surface data with an average rMSE of 143.7 mN and 216.7 mN, respectively. Normalized errors are also lower with 0.26 for the former and 0.20 for the latter. Introducing our SWEI fusion models results in performance increases for both 3D and 2D, reducing the cross-validation rMSE to 91.0 mN and 97.2 mN, respectively. When generalizing to unknown elastic properties we can further differentiate between inter- and extrapolation problems. Evaluating the pCC shown in Table 2, models trained with volumetric data but without SWEI information offer improved ability to interpolate between different elasticities compared to their 2D counterpart. Out-of-distribution generalization leads to considered increases in MAE, specifically for surface data inputs. Moreover, the extrapolation to $G_{5\%}$ is especially challenging, leading to the highest absolute and normalized errors for 2D and 3D models (see Table 2 and Fig. 6b). Phase velocity fusion provides improved generalization to the softer material and results in an error reduction of 81 % and 56 % for 2D+SWEI and 3D+SWEI, respectively.

**Table 2:** Force estimation for models tasked to generalize to unknown elastic properties: Results are compared for 2D and 3D inputs as well as our proposed method with SWEI fusion (2D+SWEI and 3D+SWEI).

		$G_i$ [$\%$]	5	7.5	10	12.5	15	17.5	20	Mean
Without SWEI	2D	rMSE	$233.5\pm 35.3$	$157.1\pm 68.1$	$138.0\pm 23.9$	$186.9\pm 8.1$	$193.8\pm 81.4$	$263.5\pm 79.6$	$295.8\pm 119.2$	$216.7\pm 60.3$
		nMAE	$0.85\pm 0.14$	$0.22\pm 0.09$	$0.15\pm 0.02$	$0.15\pm 0.01$	$0.15\pm 0.07$	$0.14\pm 0.06$	$0.14\pm 0.06$	$0.26\pm 0.26$
		pCC	$0.87\pm 0.04$	$0.81\pm 0.06$	$0.70\pm 0.07$	$0.85\pm 0.08$	0.84±0.12	0.80 ± 0.06	0.85 ± 0.06	0.78 ± 0.07
	3D	rMSE	227.1 ± 48.4	82.1 ± 11.7	69.1 ± 31.2	114.7 ± 17.5	110.5 ± 36.5	181.9 ± 109.0	180.5 ± 54.0	143.7 ± 62.3
		nMAE	0.84 ± 0.18	0.13 ± 0.03	0.08 ± 0.04	0.10 ± 0.01	0.08 ± 0.02	0.10 ± 0.07	0.08 ± 0.02	0.20 ± 0.28
		pCC	0.92 ± 0.01	0.83 ± 0.08	0.94 ± 0.01	0.93 ± 0.02	0.94 ± 0.03	0.94 ± 0.03	0.90 ± 0.03	0.89 ± 0.03
With SWEI	2D	rMSE	**45.5** ± **7.4**	52.6 ± 20.9	61.2 ± 9.3	92.0 ± 16.1	104.3 ± 23.5	152.9 ± 39.0	160.5 ± 31.0	97.2 ± 47.3
		nMAE	0.15 ± 0.02	0.08 ± 0.02	0.07 ± 0.01	0.08 ± 0.02	0.08 ± 0.02	0.08 ± 0.02	0.07 ± 0.01	0.09 ± 0.03
		pCC	0.90 ± 0.01	0.92 ± 0.08	0.94 ± 0.02	0.95 ± 0.01	0.94 ± 0.01	0.92 ± 0.04	0.94 ± 0.03	0.90 ± 0.01
	3D	rMSE	100.7 ± 15.7	**44.7** ± **25.6**	**50.8** ± **17.7**	**67.7** ± **17.1**	**101.7** ± **8.7**	**133.2** ± **26.2**	**127.3** ± **11.0**	**91.0** ± **34.7**
		nMAE	0.36 ± 0.06	0.07 ± 0.03	0.06 ± 0.02	0.06 ± 0.01	0.08 ± 0.01	0.07 ± 0.01	0.05 ± 0.01	0.11 ± 0.11
		pCC	0.96 ± 0.01	0.90 ± 0.11	0.97 ± 0.01	0.97 ± 0.02	0.95 ± 0.01	0.93 ± 0.03	0.97 ± 0.01	0.93 ± 0.02

Mean and standard deviation are given over the 7-fold cross validation where each subset represents the generalization to one unseen elasticity. Results averaged over all folds are also shown and the lowest rMSE [mN] scores are marked in bold.

### Force estimation on soft tissue

**Figure 7:**
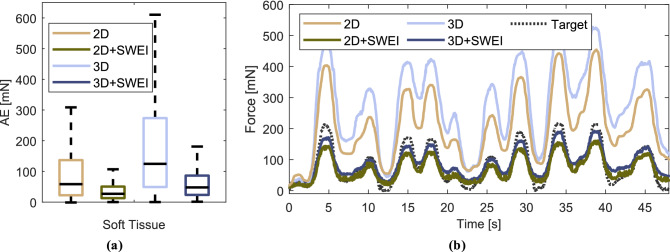
Ex-vivo Soft Tissue: **(a)** Absolute errors across all soft tissue samples plotted for each model. Accounting for sample elasticity results in superior generalization from gelatin to soft tissue. **(b)** Example force estimates for the palpation of a tissue sample for all trained models.

We ensemble the previously trained models and report the generalization from phantom to ex-vivo tissue data. The evaluation metrics for all test samples are displayed in Table 3. Absolute errors for individual estimations are also shown in the boxplot in Fig. 7a. The mean phase velocity of chicken tissue is 3.59 ± 091 m s$^{-1}$. Overall, our proposed SWEI fusion models clearly outperform 2D and 3D models without additional phase velocity input. Estimations performed on ex-vivo chicken heart tissue are feasible with an rMSE of 51.2 mN. Without SWEI, rMSE increases up to 283.15 mN with a normalized MAE as high as 0.6. An example of the resulting force estimations for all models can be seen in Fig. 7b. Models without SWEI overestimate the applied force while 2D+SWEI and 3D+SWEI models are more appropriately scaled by the phase velocity measurement.

**Table 3:** Evaluation metrics for all models trained on phantom data and tested on ex-vivo soft tissue.

Model	2D	3D	2D+SWEI	3D+SWEI
rMSE [mN]	129.5 ± 82.6	283.5 ± 212.9	**51.2** ± **24.8**	76.6 ± 35.6
nMAE	0.26 ± 0.17	0.60 ± 0.48	**0.10** ± **0.04**	0.16 ± 0.09
pCC	0.93 ± 0.03	0.94 ± 0.04	0.91 ± 0.06	**0.94** ± **0.03**

Results are averaged for all tissue samples and best results are marked bold.

## Discussion

Real-time haptic feedback during minimally invasive RAS is critical to avoid soft tissue damage and to regain the surgeons natural sense of touch^46, 47^. We show that the elastic properties of soft tissue have a strong influence in image-based force estimation. To include the biomechanical properties of soft tissue we propose a system which first, identifies the local elasticity of soft tissue with OCE and second, acquires high resolution volumetric images with OCT. We demonstrate a multi-input deep learning network which jointly processes elasticity and image information. In the following we discuss our results concerning (1) the models performance with respect to the elasticity represented in the training and evaluation data, (2) the models ability to interpolate to elasticities which are not represented in the training data as well as the impact of including elasticity sensing, and (3) the feasibility of force estimation on completely unknown soft tissue images.

Our results for models exclusively trained on a distinct gelatin gel show that force estimation on new samples is only feasibly if the elasticity is in a similar range as the training data. This is an expected results and congruent to reports in the literature that the elasticity needs to be known for accurate force estimation^48^. Although in general training and evaluation on a single bio-mechanical tissue model is feasible^21^ it is strongly limited in clinical applications. In practice, soft tissue elasticity ranges for individual tissue types, e.g., the elastic modulus for normal heart muscle is 18 ± 2 kPa and for cardiac fibrosis tissue 55 ± 15 kPa^49^. This case is represented in our data by the gelatin with a weight ratio of 5$\%$ (17.42 kPa) and 15$\%$ (56.04 kPa)^50^. Consequently, our results show that if the network is trained on healthy heart tissue and evaluated on pathological tissue the MAE could increase 20-fold (see Fig. 5a).

To alleviate this problem, we propose deep learning models that can generalize to changes in material properties. Results in Table 2 show that the fusion of SWEI provides superior performance when generalizing to new elastic properties in our phantom study. Our multi-input fusion models outperform the approaches with only image data as inputs, especially when only surface data is available. Consistent with the results shown in Fig. 6a, the largest reductions in absolute errors are achieved for the softer materials ($G_{5\%}$-$G_{10\%}$) where phase velocity measurements display low variance. For stiffer materials, the variance increases as it is more difficult to accurately detect the faster propagating waves. However, SWEI fusion is beneficial even where phase velocity estimates overlap and errors generally increase for stiffer materials due to the smaller deformations relative to the applied force. Over all elasticities unseen during training, we report a cross-validation average rMSE below 100 mN for our multi-input fusion models. In comparison, the generalization to a second synthetic material by Chua et. al. yielded an rMSE of 1865 mN for vision input data from stereographic cameras and an rMSE of 485 mN while data processing included the robot state and joint torques^18^. However, not only palpation was considered as in our case, but also pulling of the sample, making the learning task more challenging. Similarly in^51^, both interactions were regarded simultaneously and the forces along the instrument axis during the palpation task were estimated with an rMSE of 1100 mN and a pCC of 0.55. Our fusion models are also competitive with approaches that have focused on a single material only, especially for soft gelatin samples. A learning-based approach on a single heart model phantom resulted in an rMSE of 60 mN^52^. An extension of the approach was tested on two different samples from the same material and the authors reported a combined rMSE of 20 mN^27^ for the training and test set.

Our results show that elasticity information is essential when performing image-based force estimation on unknown soft tissue. It stands out, that we only train our models on gelatin phantoms and evaluate the performance on chicken heart tissue. Even though the SWEI estimates represent a simplified relationship of the complex nonlinear mechanics present in heterogeneous, anisotropic soft tissue, we show that our models are able to leverage the additional information for an improved force estimation. The networks including elasticity estimates achieve superior performance with lower rMSE and nMAE (see Table 3). Our 2D+SWEI network even outperforms our 3D+SWEI approach on soft tissue. One possible explanation is the complex structure of the soft tissue, which is anisotropic and heterogeneous. Volumetric training data from samples with a similar mechanical structure should improve performance for 3D+SWEI. Additionally, the pre-processing of the surface data also reduces the dependence on speckle properties. Regarding pCC, performance is similar for all networks, suggesting that the networks without SWEI fusion detect deformations but overestimate or underestimate the applied forces, as shown in Fig. 7a. These networks are unable to relate tissue properties and the observed deformation, as demonstrated for gelatin phantom data. Overall performance is lower compared to our cross-validation approach on gelatin phantoms, due to the uneven surface of the soft tissue and the changes in speckle properties. Our 2D+SWEI network even outperforms our 3D+SWEI approach on soft tissue which might be due to the pre-processed surface information, making it independent of speckle variations and surface characteristics. The deviation in chicken heart tissue elasticity estimates is larger than estimates from similar elasticity ranges, e.g., $G_{5\%}$ and $G_{7.5\%}$. This shows that the soft tissue elasticity is not consistent throughout the samples although visually samples look identical. Further investigating our approach on in-vivo data will be essential to study the influence of vascularization, soft tissue heterogeneity and boundary conditions regarding wave reflections. Shear wave elasticity estimates are known to be frequency dependent, as dispersion effects create a nonlinear relationship between the elasticity and frequency^53, 54^. To further refine elasticity estimates, stimulating shear waves with multiple frequencies could be implemented.

It is known that the OCE measurements for soft tissue is directly related to tissue pathology^55, 56^. Hence, we can adapt our multi-input deep learning approach to real-time classification tasks, e.g., liver fibrosis staging^19^, detecting optimal sample points for tissue biopsies or in classifying tumor tissue. One limitation of our system is the piezoelectric element which currently limits the interaction to a pushing task. Therefore, non-contact shear wave excitation via an air-pulse^34^ and the pulling task should be considered. Finally, it will be essential to translate the estimated forces into haptic feedback for the physician^57^, e.g. as kinesthetic^58^ or vibrotactile^59^ feedback.

## Conclusion

In this work, we propose image-based estimation of tool-tissue interaction forces combined with estimation of local biomechanical properties in a single modality. We present an experimental setup that enables simple and efficient data acquisition of OCE and OCT data needed for robust deep learning approaches. The conducted phantom study highlights that the influence of local elasticity cannot be neglected when estimating interaction forces. Furthermore, we show that our multi-input fusion model can generalize from phantom to soft tissue samples. Thus, a single, versatile model for image-based force estimation is feasible, which could enable real-time haptic feedback and increased autonomy in robotic-assisted interventions.

## Data Availability

The data analyzed in this study is available from the corresponding authors upon reasonable request.
